# The genome of *Gallaecimonas pentaromativorans* strain 10A, isolated from a Pacific oyster, sheds light on an environmentally widespread genus with remarkable metabolic potential

**DOI:** 10.1371/journal.pone.0334406

**Published:** 2025-10-21

**Authors:** Yasmine Gouin, Adam Wilcockson, Amy M. Chan, Curtis A. Suttle, Kevin Xu Zhong

**Affiliations:** 1 Department of Microbiology and Immunology, The University of British Columbia, Vancouver, British Columbia, Canada; 2 Department of Earth, Ocean and Atmospheric Sciences, The University of British Columbia, Vancouver, British Columbia, Canada; 3 Department of Botany, The University of British Columbia, Vancouver, British Columbia, Canada; 4 Institute for Oceans and Fisheries, The University of British Columbia, Vancouver, British Columbia, Canada; Institute of Biological Sciences, University of the Philippines Los Baños, PHILIPPINES

## Abstract

Bacteria in the genus *Gallaecimonas* are known for their ability to breakdown complex hydrocarbons, making them of particular ecological and biotechnological significance. However, few species have been isolated to date, and their ecological distribution has yet to be examined. Here, we report a novel strain of *G. pentaromativorans*, designated as strain 10A, which was isolated from a Pacific oyster (*Magallana gigas*, a.k.a. *Crassostrea gigas*) collected from a farm experiencing a mass mortality event in British Columbia (BC), Canada. *Gallaecimonas pentaromativorans* strain 10A is a rod-shaped, motile bacterium and has a circular genome of 4,322,156 bp encoding 3,928 protein-coding sequences (CDS). Phylogenetic analysis showed that strain 10A is closely related to members of *G. pentaromativorans.* Like other *Gallaecimonas* members, strain 10A is predicted to harbor specific pathways involved in degrading xenobiotic compounds including polycyclic aromatic hydrocarbons (PAHs), producing biosurfactants, and assimilating nitrate and sulfate; however, it is uniquely equipped with an additional 166 genes belonging to 147 protein families, including a putative *higB*-*higA* that likely contributes to enhanced stress response. Strain 10A also possesses Clustered Regularly Interspaced Short Palindromic Repeat (CRISPR) and CRISPR-associated (Cas) system (CRISPR-Cas), prevalent in *Gallaecimonas* (detected in three out of four species), implying a potential defense mechanism against exogenous mobile genetic elements such as plasmids and viruses. We also mined publicly available databases to establish the widespread distribution of bacteria in the genus *Gallaecimonas* in seawater, sediments, and freshwater across latitude, suggesting its versatility and importance to environmental processes. Ultimately, this study demonstrates that the genome of *G. pentaromativorans* strain 10A, isolated from a Pacific oyster, may encode a suite of putative functions, including xenobiotic breakdown, biosurfactant production, and CRISPR-Cas defense. This plasticity and breadth in metabolic function help to explain the cosmopolitan distribution of members of this genus.

## Introduction

Bacteria in the genus *Gallaecimonas* (class *Gammaproteobacteria*) are known for their ability to breakdown xenobiotic compounds, including polycyclic aromatic hydrocarbons (PAHs) [1–5]. Consequently, they have become important allies in bioremediation, particularly in the degradation of oil-derived compounds and other xenobiotic pollutants, such as toluene, fluorobenzoate, and chlorobenzene [1–5]. In addition, they can also produce biosurfactants (e.g., glycolipids, lipopeptides, polysaccharide-protein complexes, phospholipids, fatty acids, and neutral lipids), natural surface-active molecules that facilitate processes such as emulsification, dispersion, and solubilization, supporting applications across environmental, pharmaceutical, and agricultural sectors [2]. Furthermore, one *Gallaecimonas* species has demonstrated antimicrobial activity against the bacterial pathogen *Vibrio harveyi* through the production of cyclic peptides, specifically diketopiperazines [6].

Despite their ecological and industrial importance, there are only four described isolates in the genus *Gallaecimonas*, each belonging to a different species: *G. pentaromativorans* [1], *G. xiamenensis* [3], *G. mangrovi* [4], and *G. kandeliae* [5]. Members of the genus have been reported from mid-latitude intertidal sediments and seawater, as well as in symbiotic associations with plant hosts, including the mangrove *Kandelia obovate* [1,3–5]. In spite of these discoveries, the number of cataloged *Gallaecimonas* species remains limited, and their ecological distribution is largely unexplored.

Here, we genomically characterize *G. pentaromativorans* strain 10A, which we isolated from a Pacific oyster during a mass mortality event of oysters in British Columbia (BC), Canada. This study benchmarks the genomic architecture and genetic capabilities of strain 10A against those of other members within the genus *Gallaecimonas*. Additionally, by interrogating publicly accessible datasets of 16S ribosomal RNA (rRNA) gene sequences, we elucidate the global distribution of members of the genus *Gallaecimonas*. Overall, this research highlights the genetic potential of strain 10A, hinting at possible ecological and biotechnological applications.

## Materials and methods

### Bacterial isolation and culture conditions

Pacific oysters were collected from a tray at an aquaculture facility in the Baynes Sound area (49.5078° N, 124.8272709° W), BC, Canada, during a mortality event in July 2020. These oysters exhibited varying degrees of mortality (non-gaping to gaping, dead); shell lengths ranged from 5 to 7 cm. The oysters were opened to harvest tissue samples within 1 hour of collection. Samples were placed into sterile Whirl-Pak sample bags and immediately flash frozen in a liquid nitrogen (LN_2_) dry shipper, where they remained frozen in LN_2_ vapors for one week, then stored at −80 ºC.

*Gallaecimonas pentaromativorans* strain 10A was isolated from tissue collected from a non-gaping oyster. Approximately 1.5 grams of frozen oyster tissue was aseptically transferred to a sterile 50 mL Falcon tube, thawed on ice, and then combined with 1.5 mL of sterile F/2 seawater media [7]. This mixture was gently vortexed to create a homogenate, from which the bacterium was isolated.

To prepare spread-plate cultures, 50 µL of the oyster homogenate was pipetted onto CPM-24 plates (0.05% Difco Casamino Acids, 0.05% Difco Peptone, 1% Fisher Scientific purified agar; prepared with 24 practical salinity units (PSU) seawater) [8,9] and MLB-24 plates (CPM-24 with additional 0.05% Yeast Extract and 0.3% glycerol) [8,9] and sterile 10-uL plastic inoculating loops were used to evenly spread the sample over the plate surface until all liquid was adsorbed. After incubating at room temperature (ca. 21ºC) for about a week, a colony was cleanly picked and re-streaked four times onto CPM-24 plates, using a single well-separated colony each time to obtain axenic clonal cultures. Subsequently, the purified isolate was routinely cultured with MLB media prepared with 24 PSU seawater (MLB-24); stock cultures were preserved in 20% (v/v) glycerol and stored at −80°C.

The soft agar motility test [10] was performed by stabbing a tube of MLB-24 soft agar (0.6% agar) with cells picked from a colony grown on MLB-24 agar and monitoring growth away from the stab line after 2–3 d.

### Transmission electron microscopy (TEM)

To assess the morphology of strain 10A, a few colonies from a culture grown on MLB-24 agar were resuspended in 0.2 µm-filtered seawater, then fixed with 0.2 µm-filtered EM grade glutaraldehyde (25%) to achieve a final concentration of 1% glutaraldehyde. The fixed sample was adsorbed to the shiny side of a formvar-carbon 400 mesh copper grid (Cat# 01754-F, TedPella, CA) for 5 min. Excess sample was wicked away with filter paper, and the grid was then stained with 2% uranyl acetate for 30 s. Excess stain was wicked away, and the grid was allowed to air dry at room temperature for at least 5 minutes before visualization at 120 kV on a Tecnai Spirit transmission electron microscope.

### Genomic DNA extraction, sequencing, and genome assembly

To prepare a sample for whole genome sequencing, strain 10A cultures grown on MLB-24 agar plates for 2 d were used to inoculate two 50-mL Falcon tubes, each with 20 mL of MLB-24 broth. The cultures were grown in an orbital shaker at 24ºC and 150 rpm for 2 d. Cells were harvested by pelleting in a Beckman Allegra X-22R Benchtop centrifuge with a swinging bucket rotor at 3730 x *g* and 10°C for 15 min. The cell pellets were resuspended in approximately 4 mL of sterile F/2 seawater media (24 PSU) and dispensed into two 2 mL cryovials. Cells were pelleted in a Beckman Allegra X-22R centrifuge with a fixed-angle rotor at 9000 x *g* and 10°C for 5 min, then frozen using dry ice after removal of the supernatant.

The bacteria were submitted to the Microbial Genome Sequencing Center (MiGS) at the University of Pittsburgh (Pittsburgh, PA) for genomic DNA extraction (Zymo fungal/bacterial DNA miniprep kit; Zymo Research, Irvine, CA), and hybrid assembly sequencing (Small Nanopore Combo package). The Illumina sample was prepared using the Illumina DNA library prep kit (Illumina, Inc., San Diego, CA) and sequenced on an Illumina NextSeq2000 instrument with 151-bp paired-end chemistry, generating 4,117,269 Illumina short-reads. The Nanopore sample was prepared using the Oxford Nanopore Technologies (ONT, UK) ligation sequencing kit and sequenced on a MinION instrument using an R9 flow cell (R9.4.1), with base calling performed using ONT Guppy v.4.2.2, yielding 144,908 ONT long-reads. Adapters and low-quality reads were trimmed using bcl2fastq v.2.19.0 [11] and porechop v.0.2 [12] for Illumina and ONT sequences, respectively. Hybrid assembly with Illumina and ONT reads was performed using Unicycler v.0.5 [13]. The integrity of the bacterial genome was checked using CheckM v.1.0.18 [14].

### Taxonomical classification and phylogenetic analysis

The taxonomic identity of strain 10A was determined using currently well-established pipelines such as Kaiju v.1.8.2 [15], CAT v.5.2.3 [16], MetaErg v.1.2.3 [17], the NCBI Prokaryotic Genome Annotation Pipeline (PGAP) v.6.0 [18], and GTDBtk v.2.3.2 [19] with default parameters. Once identified as a member of the genus *Gallaecimonas*, phylogenetic placement was done by whole-genome phylogeny using GTDBtk v.2.3.2 [19] and publicly available genomes of *Gallaecimonas* spp. from the Genome Taxonomy Database (GTDB, v.217). Phylogenetic analysis of the 16S rRNA gene was also done on complete 16S rRNA sequences for *Gallaecimonas* spp. from the SILVA rRNA gene database v.138.1 [20]. These sequences, along with that of strain 10A, were aligned using MAFFT v.7 [21] with default parameters. The alignments were then trimmed using Gblocks [22] with default parameters. The maximum-likelihood tree was constructed using PhyML 3.0 [23] with a bootstrap parameter of 100, and the best model (TN93) was determined using MEGA11 [24]. The tree was visualized with Geneious Prime v.2023.2.1.

### Gene prediction, annotation, and visualization

The genome was annotated using the NCBI Prokaryotic Genome Annotation Pipeline (PGAP) v.6.0 [18] with default parameters. Subsequently, the PGAP-predicted genes were annotated using eggNOG-mapper v.2 [25] with default parameters based on ortholog mapping against the eggNOG (Evolutionary genealogy of genes: Non-supervised Orthologous Groups) database, providing functional annotations such as seed ortholog (i.e., gene family), eggNOG OGs, COG category, PFAMs (i.e., protein family), EC, GO terms, CAZy, BiGG Reaction, BRITE, and KEGG profiles [26] including KEGG Ortholog (ko), KEGG Pathway, KEGG Module, KEGG Reaction, KEGG rclass, and KEGG TC. In addition, MetaErg v.1.2.3 [17] was also employed using default parameters to provide supplementary annotation, including MetaCyc pathways. Genome features were visualized using Proksee [27], and their KEGG profiles were visualized using FuncTree v.0.8.4 [28]. CRISPR arrays (including the CRISPR repeat and spacer sequences), as well as the Cas protein clusters, were identified using CRISPRCasFinder v.4.3.2 [29] with default settings. To reaffirm the hydrocarbon-degrading potential of strain 10A, its translated protein sequences were queried against the CANT-HYD [30] (https://github.com/dgittins/CANT-HYD-HydrocarbonBiodegradation) and HADEG [31] (https://github.com/jarojasva/HADEG) databases using each pipeline’s default settings.

### Comparative analysis of *Gallaecimonas* genomes

The genome of strain 10A was compared with genomes from NCBI for the following isolates of *Gallaecimonas* spp.: *G. xiamenensis* strain 3-C-1 [3,32], *G. pentaromativorans* strain CEE_131 (Leibniz Institute DSMZ culture collection ID: DSM 21945) [1], *G. mangrovi* strain HK-28 [4], and *G. kandeliae* strain Q10 [5]. A pan-genome analysis and visualization were performed using PGAP v.6.0 [18], eggNOG-mapper v.2 [25], R packages ggplot2 v.3.4.4 [33] and ggvenn v.0.1.10 [34], and Proksee [27] with default settings. Prior to the analysis, to ensure consistent gene calling and annotation, the genomes were re-annotated using PGAP v.6.0 and eggNOG-mapper v.2 following the methodologies outlined above in “Gene prediction, annotation and visualization”. PGAP-predicted genes were grouped into different gene families (i.e., seed_ortholog) based on eggNOG-mapper annotations to determine shared genes across the different genomes. The core genomic features, including gene families (i.e., seed_ortholog), protein families (i.e., PFAMs), CAZy, COG profiles, EC, eggNOG Ogs, GO terms, KEGG ko profiles, and KEGG Module profiles, were defined by the presence of identical IDs across the analyzed genomes. Comparisons of these genomic features were analyzed and visualized using R packages ggplot2 v.3.4.4 and ggvenn v.0.1.10. Key functional pathways found in strain 10A were depicted using BioRender (https://www.biorender.com).

### Ecological distribution of *Gallaecimonas*

The ecological distribution (presence/absence and relative abundance) of *Gallaecimonas* spp. was investigated based on 16S rRNA gene sequences between 2003 and 2019 obtained from the Global Biodiversity Information Facility (GBIF) database [35] (S5 Table; https://www.gbif.org/). Using the coordinates of the sampling sites provided in occurrence data, the global distribution was visualized using R packages ggplot2 v.3.4.4 [33], sf v.1.0−14 [36], and maps v.2.3−2 [37]. Additionally, the proportion of the number of 16S rRNA gene sequences assigned to *Gallaecimonas* spp. across different environments was determined through the habitat types of the sampling sites.

## Results

### Isolation and visualization of strain 10A

*Gallaecimonas* *pentaromativorans* strain 10A was isolated from an oyster aquaculture farm experiencing a mortality event. The cultures were purified by streaking repeatedly on solid media. When grown on MLB-24 plates, at 21°C for 3–4 days, colonies were circular, convex, colorless and translucent, and 0.5- to 1- mm in diameter. Colonies that were well separated from each other could be as large as 3 mm in diameter in 3-week-old cultures; these colonies appeared to be less translucent and whiter in color. Strain 10A cells stained with 2% uranyl acetate and visualized with transmission electron microscopy (TEM) were rod-shaped, approximately 0.5-μm wide, and up to 2-μm long, and some appeared to possess a polar flagellum. A soft agar motility stab test was done by stabbing a tube of soft agar with cells. Diffuse waves and swirls of cell growth away from the initial stab line confirmed that strain 10A was motile.

### Genomic characterization of the strain 10A

**Fig 1:**
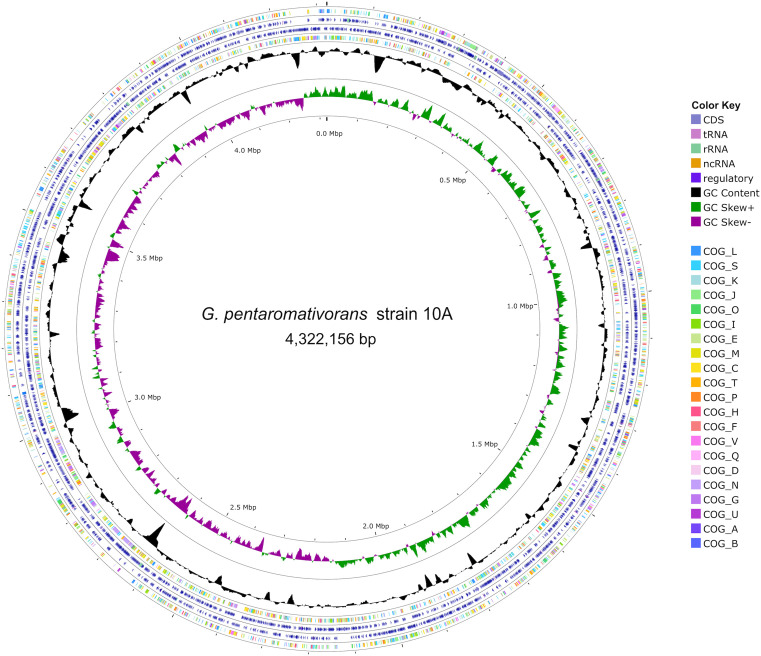
The complete genome of the *Gallaecimonas pentaromativorans* strain 10A. The outermost to innermost rings of the map represent the following: clusters of orthologous genes (COGs) functional categories for forward strand coding sequences; forward strand sequence features; reverse strand sequence features; COGs functional categories for reverse strand coding sequences; black ring shows GC content; and GC skew, with the green and purple bands representing positive and negative values, respectively.

Hybrid sequencing using Illumina and Nanopore technologies allowed for assembling the genome sequence of strain 10A, which consisted of a circular chromosome of 4,322,156 bp with an average GC-content of 58.3% (Fig 1). The integrity of the bacterial genome was validated using CheckM v.1.0.18, which showed that the genome was 99.82% complete and had 0.00% contamination compared to 899 reference gammaproteobacterial genomes in the database. Annotation using the National Centre for Biotechnology Information (NCBI) Prokaryotic Genome Annotation Pipeline (PGAP) v.6.0, revealed that strain 10A contains genes coding for 18 rRNA (six of each 5S, 16S, and 23S rRNAs), 86 tRNAs, and four ncRNAs, as well as 3,928 protein-coding sequences (CDS). These CDS belong to 2734 protein families (i.e., PFAMs) and encode proteins involved in a wide array of biological processes integral to metabolic and regulatory functions. Putatively, they include 21 Clusters of Orthologous Genes (COGs), 891 enzymes, 2,564 Gene Ontology profiles (GO), 1,359 KEGG Ortholog (ko) profiles, as well as 112 KEGG and 439 MetaCyc pathways (Fig 1; S1-S2 Figs).

**Fig 2:**
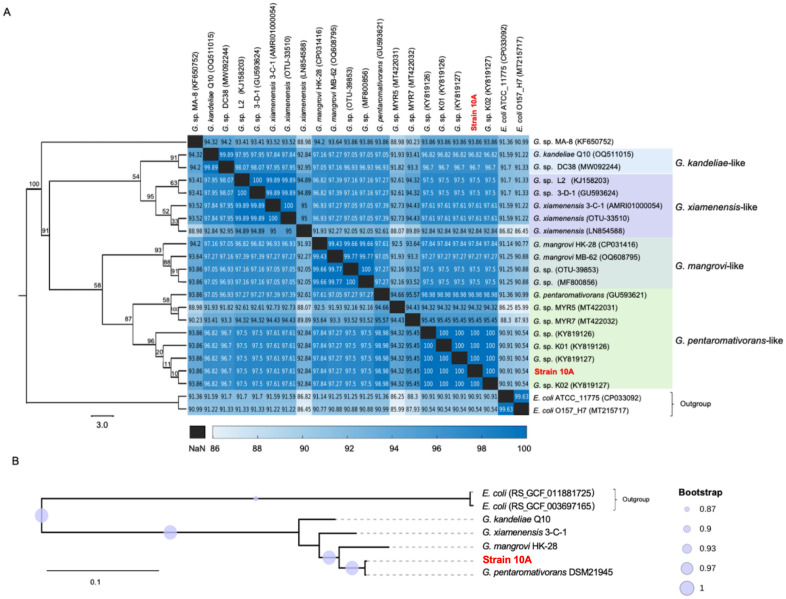
Phylogenetic relationship among DNA sequences from *Gallaecimonas* spp. **(A)** Phylogenetic relationship of the 16S rRNA gene sequences from strain 10A and other *Gallaecimonas* spp. found in the SILVA rRNA gene database v.138.1. The maximum-likelihood tree was built using 100 replicates and rooted with sequences from two strains of *Escherichia coli* (NCBI accession numbers: CP033092.1 and MT215717.1) as an outgroup. The value in the heatmap associated with the phylogenetic tree represents the average nucleotide identity (ANI) of 16S rRNA gene sequences between two strains on each of the x and y axes. **(B)** Phylogenomic tree of bacteria in the genus *Gallaecimonas* based on data in NCBI Reference Sequence Database (RefSeq, v.216). The tree was built based on the overall genome sequences and rooted with two genome sequences from *Escherichia coli* (GTDBtk accession numbers: RS_GCF_011881725.1 and RS_GCF_003697165.2) as an outgroup. *Gallaecimonas pentaromativorans* strain CEE_131 is also known as *G. pentaromativorans* strain DSM 21945.

Phylogenetic analysis of the 16S rRNA gene sequences available in the SILVA rRNA gene database v.138.1, showed that strain 10A clustered with members of *G. pentaromativorans* with a high bootstrap value (87/100; Fig 2A). Phylogenomic analysis based on whole genomes of *Gallaecimonas* spp. are consistent with strain 10A being closely related to *G. pentaromativorans* strain CEE_131 (Leibniz Institute DSMZ culture collection ID: DSM 21945; Fig 2B). Specifically, the average nucleotide identity (ANI) between the genomes of strain 10A and CEE_131 is 98.98%, and their 16S rRNA gene sequences share 97.3% identity (Fig 2), consistent with their classification as members of the same species. This classification is further supported based on overall genomic features, which place strain 10A in the genus *Gallaecimonas* (using NCBI PGAP v.6.0, GTDBtk v.2.3.2, MetaErg v.1.2.3, and CAT v.5.2.3) or in the species *G. pentaromativorans* (using Kaiju v.1.8.2).

### Comparative analysis of *Gallaecimonas* genomes

**Fig 3:**
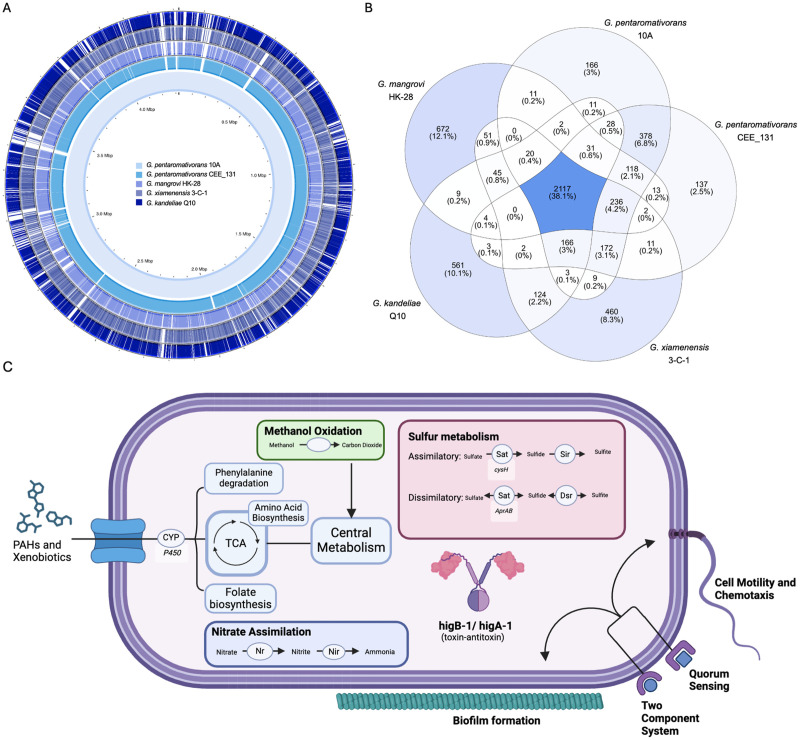
Comparative genomics among strains of *Gallaecimonas* spp. **(A)** Comparative map depicting genomic arrangements of four other isolates of *Gallaecimonas* spp. to strain 10A. **(B)** Venn diagram illustrating the distribution of shared and unique genes among the five strains of *Gallaecimonas* spp. **(C)** Key functional pathways found in *G. pentaromativorans* strain 10A, including PAHs and xenobiotic degradation, methanol oxidation, nitrate assimilation, sulfur metabolism, higB-1/higA-1 toxin-antitoxin system, and biosurfactant-producing pathways. *Gallaecimonas pentaromativorans* strain CEE_131 is also known as *G. pentaromativorans* strain DSM 21945.

The shared genomic features among isolates of *Gallaecimonas* spp. were investigated by a pan-genomic analysis of *G. xiamenensis* strain 3-C-1, *G. pentaromativorans* strain CEE_131 (DSM 21945), *G. mangrovi* strain HK-28, *G. kandeliae* strain Q10, and *G. pentaromativorans* strain 10A (Fig 3A-3B). These five isolates revealed 5562 genes; 38.1% were designated as “core” genes because they are shared by all the isolates, while the remainder are distributed across species. The “core” genes, which represent 60.45 to 66.95% of the genes in each species, were integrated into 1677 gene families (i.e., seed_ortholog in eggNOG-mapper annotation), 1586 KEGG ko profiles, 230 KEGG modules, and 401 KEGG pathways, as predicted using eggNOG-mapper (Fig 3B; S1-S2 Figs; S2 Table). These “core” genes are involved in metabolic processes such as cell growth and death, binary fission, the citrate cycle, the metabolism of amino acids (e.g., *HisD, IlvC, SerA, Tdh),* and peptidoglycan synthesis *(*i.e., *murABCDEF, Pbp* genes*, Ddl)*. Notably, several highly conserved pathways, with over 50% coverage for both KEGG module and pathway profiles, entail two-component systems crucial for environmental processing: osmotic stress response (*MtrB-MtrA)*, cell fate control (*PleC-PleD)*, type 4 fimbriae synthesis *(PilS-PilR)*, cPHB biosynthesis (*AtoS-AtoC)*, membrane lipid fluidity *(DesK-DesR)*, capsule synthesis *(ResC-ResD-ResB)*, hexose phosphate uptake (*UhpA-UhpB)*, and cell wall metabolism *(VicK-VicR)* (S1 and S2 Figs).

Despite the conserved genetic nature among isolates of *Gallaecimonas* spp., each member has between 137 and 672 characteristic genes, exhibiting inter-species genomic variability (Fig 3B; S2 Table). For instance, *G. xiamenensis* strain 3-C-1 bears 460 distinct genes (13.5% of genes in strain 3-C-1), among which are those related to copper tolerance, such as multicopper oxidase, *CusS-CusR* two-component system, and divalent heavy-metal cations transporters (*Zip*). In contrast, *G. kandeliae* strain Q10 possesses 561 specific genes (18.1% of its genes), including those encoding zinc-binding dehydrogenase (ADH_N, ADH_zinc_N, ADH_zinc_N_2) and AMP-dependent synthetase and ligase (*AMP-binding, AMP-binding_C*). Similarly, the *G. mangrovi* strain HK-28 carries 672 different genes (17.7% of its genes), encompassing those involved in the ABC transporter superfamily (*ABC_tran, oligo_HPY*), and guanosine tetraphosphate metabolism (*RelA, SpoT*). Even *G. pentaromativorans* strain CEE_131 (DSM 21945) exhibits 137 specific genes (4% of its genes), including cysteine-rich domain-containing proteins (CCG) and Type I restriction enzyme R protein N terminus (*HSDR_N, HSDR_N_2*).

Intraspecies conservation of genes is evident in *G. pentaromativorans*, represented by strains CEE_131 (DSM 21945) and 10A. These isolates share 378 genes, representing 40 KEGG ko profiles, that differ from those found in other *Gallaecimonas* spp. (S2 Fig; S2 Table). Of note are genes putatively encoding aldehyde dehydrogenase (e.g., *badH)*, thiamine pyrophosphate enzymes (e.g., *poxB)*, alpha/beta hydrolases, SMART protein phosphatase 2C domain proteins, and fatty acid desaturase (S2 Table).

Inter- and intra-species variation among isolates of *Gallaecimonas* spp. is evident through analysis of strain 10A. In comparison to other isolates of *Gallaecimonas* spp., including *G. pentaromativorans* strain CEE_131 (DSM 21945), strain 10A uniquely possesses 166 genes (4.2% of genes in strain 10A), integral to 147 protein families, 14 ko profiles and 1 KEGG pathway (Fig 3B; S2 Fig; S2 Table). These genes are predicted to encode for proteins involved in diverse biological functions, including DNA methylation (K00590), heme export for c-type cytochrome biogenesis (*CcmD*), flagellar transcriptional activation (*FlhC*), peptidase activity (K06992), capsule biosynthesis (*hipA*), and hemolysin activity (*shlB).* Moreover, a putative higB-1/higA-1 toxin/antitoxin (TA) system (e.g., K21498) was found in strain 10A, but was absent in other *Gallaecimonas* strains (Fig 3B-C). In addition, a KEGG pathway consisting of (i) a two-component system (ko02020), (ii) quorum sensing (ko02024), (iii) biofilm formation (ko02026), and (iv) flagellar assembly (ko02040), was identified in strain 10A, but not in other *Gallaecimonas* strains (Fig 3B-C). While not unique to strain 10A, of particular note are vital marker genes involved in methanol oxidation (e.g., *pqq, xoxF, mxat*), as well as complete pathways for assimilatory and dissimilatory sulfate reduction (M00176, M00596) and nitrate assimilation (e.g., *nasA*).

Further, analysis using eggNOG-mapper and FuncTree (based on KEGG profiles) revealed that *G. pentaromativorans* strain 10A encodes a broad repertoire of genes putatively associated with the metabolism of xenobiotic compounds, including PAHs (e.g., naphthalene) and other environmentally relevant pollutants such as benzoate, ethylbenzene, caprolactam, chloroalkanes, atrazine, styrene, nicotinate, and nicotinamide (Fig 3; S3 Fig; S1 Table). These sequences are predicted to include genes encoding enzymes involved in aromatic ring cleavage (*catA, hmgA*), processing of downstream intermediates such as lactones and keto acids (*uptA, ycgM,* and unnamed genes encoding dienelactone hydrolase), detoxification (*gstA*), and transport of aromatic intermediates (*benE*), consistent with metabolic versatility in processing aromatic and xenobiotic compounds. To complement these predictions, we further investigated the genome of strain 10A for genes involved in hydrocarbon degradation, given that PAHs represent a major class of aromatic hydrocarbons. A homology-based search against the curated hydrocarbon degradation gene database [30,31] identified several candidate genes (S3 Table). Among these, *pcaJ* (3-oxoadipate CoA-transferase subunit B) and *pcaF* (β-ketoadipyl-CoA thiolase) are linked to the β-ketoadipate pathway, which funnels PAH-derived intermediates into central metabolism [38]. Other identified genes, including *alkB* (alkane 1-monooxygenase) and *alkJ* (alcohol dehydrogenase), are associated with aliphatic hydrocarbon metabolism, while *ahpF* (oxidative stress defense), *phaZ* (polyhydroxyalkanoate depolymerase), and *lipA* (lipase A) indicate auxiliary roles in hydrocarbon mobilization, storage, and stress adaptation [39–42].

**Fig 4:**
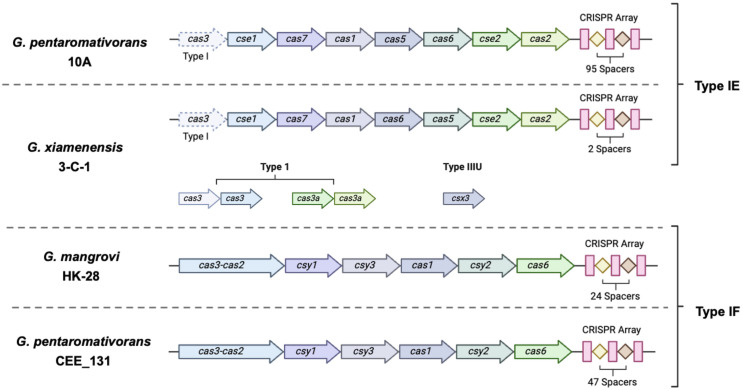
Detection of CRISPR-Cas systems in *Gallaecimonas* spp. Operon organization of different CRISPR-Cas systems detected in genomes of *Gallaecimonas* spp., annotated with the clustered regularly interspaced short palindromic repeats (CRISPR), spacers, and CRISPR-associated (Cas) proteins. Annotations are based on sequence similarities to known Cas proteins using HMM protein profiles and identified using CRISPRCasFinder. *Gallaecimonas pentaromativorans* strain CEE_131 is also known as *G. pentaromativorans* strain DSM 21945.

In addition, we identified the CRISPR-Cas systems, a well-known prokaryotic defense system against exogenous plasmids and viruses [43], in the genome of *G. pentaromativorans* strain 10A, as well as in three other *Gallaecimonas* members, spanning three out of four *Gallaecimonas* species (Fig 4; S4-S8 Figs). *Gallaecimonas mangrovi* strain HK-28 and *G. pentaromativorans* strain CEE_131 (DSM 21945) have identical *Cas*-TypeIF clusters, comprising *cas3-cas2, csy1, csy3, cas1, csy2,* and *cas6* (Fig 4; S4-S6 Figs). Furthermore, four Cas clusters were identified in *G. xiamenensis* strain 3-C-1 (Fig 4; S7 Fig). Of note, two distinct Type I Cas clusters, featuring cas3a and cas3, respectively, and a Cas-TypeIIIU cluster containing *Csx3*-TypeIIIU were detected. In addition, a TypeIE CRISPR-Cas system was also found in both *G. xiamenensis* strain 3-C-1 and strain 10A and was characterized by a *cas2, cas1, cas6, cas5, cas7, cse2, cse1*, and an accessory c*as3-*TypeI (Fig 4; S7-S8 Figs). Moreover, three CRISPR arrays (i.e., including the CRISPR repeat and spacer sequences), two with one spacer, and one with 95 spacers, were detected in the genome of strain 10A (S8 Fig; S4 Table). In summary, between one and ten CRISPR arrays were detected in all five *Gallaecimonas* genomes (Fig 4; S4-S8 Figs), although no *Cas* genes were identified in *G. kandeliae* strain Q10.

### Global distribution of *Gallaecimonas*

**Fig 5:**
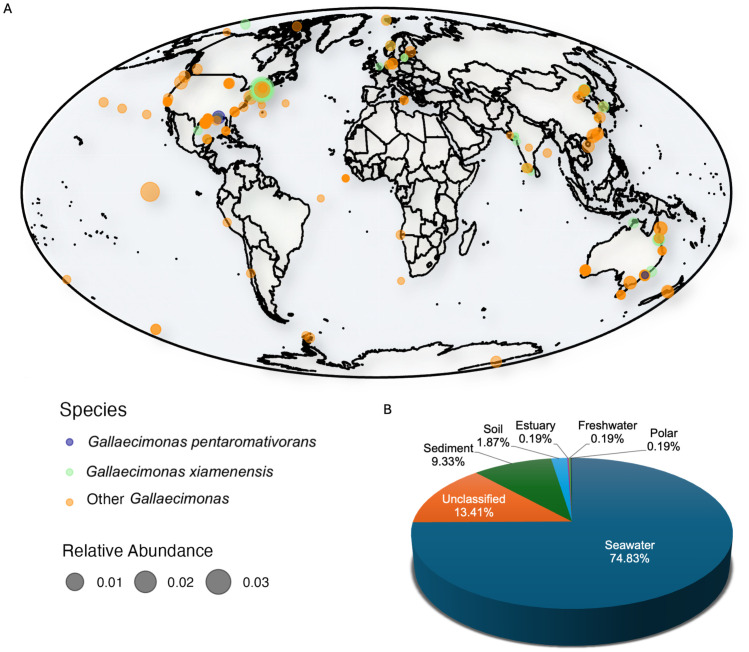
Distribution of *Gallaecimonas* across locations and habitats. **(A)** The global distribution of 16S rRNA gene sequences assigned to *Gallaecimonas* spp. was assessed using 536 sequences sourced from the GBIF Database. Orange and green indicate sequences assigned to *G. pentaromativorans* and *G. xiamenensis*, respectively, while “Other *Gallaecimonas*” comprises *G. kandeliae*, *G. mangrovi* and unclassified *Gallaecimonas* spp. The circle size represents the relative abundance of sequences assigned to *Gallaecimonas* spp. within the prokaryotic community. The base map was generated using R package maps v.2.3−2, with internal boundary data derived from Natural Earth (https://www.naturalearthdata.com) via the maps package. **(B)** The proportion of 16S rRNA gene sequences assigned to *Gallaecimonas* spp. across environments. Seawater, estuary, and freshwater correspond to water samples from the respective environments. Polar represents the cryosphere and sediment includes samples from marine (e.g., pelagic sediment cores, benthic continental shelf), rivers, wetlands, saltmarshes and tidal mudflats.

Examination of the Global Biodiversity Information Facility (GBIF) 16S rRNA gene database [35] revealed sequences for *Gallaecimonas* spp. from a wide range of habitats spanning from polar regions to equatorial zones (Fig 5; S5 Table). In total, 536 16S rRNA gene OTUs assigned to *Gallaecimonas* were detected in the database*.* Of these 536 OTUs, 37.18% were resolved to species, with *G. pentaromativorans* representing 0.42% and *G. xiamenensis* representing 36.76% of the total. The remaining 62.82% of sequences were assigned to other *Gallaecimonas* spp., including *G. kandeliae*, *G. mangrovi*, and unclassified *Gallaecimonas,* including *Gallaecimonas* sp. SSL4–1, *Gallaecimonas* sp. MA-8, and *Gallaecimonas* sp. L2 (Fig 5A; S5 Table). Notably, most *Gallaecimonas*-containing samples found in GBIF were from marine environments (74.83%), including the pelagic ocean, saltmarshes, beaches, coral reefs, aquaculture ponds, and tidal flats (Fig 5B; S5 Table). Nonetheless, the footprint of *Gallaecimonas* also extends to other environments, including sediments (9.33%), forest soil (1.87%), estuaries (0.19%), rivers (0.19%), and polar systems (0.19%), with the remainder to be classified (13.41%) (Fig 5B). Overall, the relative abundance of *Gallaecimonas* 16S rRNA gene sequences in these samples (n = 536) ranged between 0.000048 and 3.99% (Fig 5A; S5 Table).

## Discussion

Bacteria in the genus *Gallaecimonas* have important industrial and ecological roles as biosurfactant producers and oil degraders [1,2]. Here, we report on *G. pentaromativorans* strain 10A, the first isolate of *Gallaecimonas* spp. to be reported from an animal host, the Pacific oyster (*Magallana gigas*, also known as *Crassostrea gigas*). The sequencing of strain 10A provides the first complete genome for *G. pentaromativorans*, significantly expanding the genomic resources for this genus. This discovery, together with comparative genomic and biogeographic analyses, offers new insights into the ecological distribution and functional potential of *Gallaecimonas*.

### Strain 10A has genetic potential for PAH and xenobiotic degradation

Polycyclic aromatic hydrocarbons (PAHs) are ubiquitous, recalcitrant pollutants of natural and anthropogenic origin, with high potential for bioaccumulation and carcinogenesis [44,45]. They belong to a broader class of compounds known as xenobiotics, which includes primarily synthetic, but also some naturally occurring, foreign chemicals, such as pesticides and industrial solvents, that are often resistant to biodegradation and can persist in marine ecosystems [46–49]. Given the persistence and toxicity of PAHs and other xenobiotic compounds in marine environments, the microbial capacity to degrade or biotransform these pollutants is particularly valuable [44–48,50,51]. *Gallaecimonas pentaromativorans* strain 10A possesses putative genes (e.g., *benE, catA, gstA, hmgA, pcaF, pcaJ, uptA, ycgM*) implicated in the breakdown of PAHs (e.g., naphthalene) and other xenobiotic compounds (e.g., ethylbenzene, caprolactam, chloroalkanes, atrazine, styrene, nicotinate, benzoate, and nicotinamide), suggesting metabolic versatility with potential applications in bioremediation (S3 Fig; Fig 3C; S1 and S3 Tables). Although canonical PAH-initiating dioxygenases (e.g., *nahA*) are yet to be identified, strain 10A is predicted to harbor downstream catabolic genes encoding enzymes such as intradiol ring-cleavage dioxygenase (*catA*-like), homogentisate 1,2-dioxygenase (*hmgA*), dienelactone hydrolase (DLH), 2-keto-4-pentenoate hydratase (*uptA, ycgM*), as well as 3-oxoadipate CoA-transferase subunit B (*pcaJ*) and β-ketoadipyl-CoA thiolase (*pcaF*), which mediate the cleavage and further metabolism of PAH-derived intermediates such as catechol and protocatechuate via the meta- and ortho-cleavage pathways (S1 and S3 Tables) [38,52–57]. This genomic profile of strain 10A is consistent with other members of the genus, including *G. pentaromativorans* strain CEE_131 (DSM 21945), which has been experimentally reported to degrade high-molecular-weight PAHs such as pyrene and benzo[a]pyrene [1]. Notably, similar to strain 10A, strain CEE_131 (DSM 21945) also lacks the canonical ring-hydroxylating dioxygenases (e.g., *nahA*-like genes) that typically initiate aerobic PAH degradation (S1–S3 Tables) [54,57], suggesting that PAH metabolism in *Gallaecimonas* spp. may proceed through alternative or yet-uncharacterized mechanisms, as has been observed in other PAH-degrading microbes [57–59]. Consequently, while strain 10A encodes multiple downstream catabolic genes (e.g., *catA, uptA, ycgM, pcaJ, pcaF, hmgA*), further experimental validation is needed to confirm its ability to degrade PAHs. Nevertheless, our genomic analysis of strain 10A highlights its genetic potential as a bioremediation agent in contaminated environments, particularly for PAHs, which are prevalent in coastal marine environments due to anthropogenic inputs and tend to accumulate in filter-feeding organisms such as oysters [44–48,50,60–64].

### Strain 10A is a potential candidate for a biosurfactant producer

Biosurfactants play a crucial role in emulsifying hydrophobic substrates, thereby enhancing nutrient accessibility and promoting microbial growth, which is particularly important in ecological niches [2,65]. In the genome of strain 10A, we detected genes encoding enzymes involved in biosurfactant production, including those for the synthesis of phospholipid (e.g., *plsB*, *plsC*, *plsX*, *plsY*), fatty acids (e.g., *accA*, *accB*, *accC*, *accD*, *bioA*, *bioB*, *bioC*, *bioD*, *bioF*, *bioH*, and unnamed genes encoding fatty acid desaturase), and glycolipids (e.g., *pelA* and unnamed genes encoding glycosyltransferase family 1 and 4 proteins) (S1–S2 Tables), highlighting the diverse biosynthetic capacities of strain 10A for producing critical components like phospholipids, fatty acids, and glycolipids that facilitate ecological interactions and have potential industrial applications.

In addition to biosynthetic genes, strain 10A is also predicted to encode a suite of regulatory pathways consisting of (i) two-component systems (e.g., *DesK-DesR*, *PilS-PilR, MtrB-MtrA, PleC-PleD, AtoS-AtoC, ResC-ResD-ResB, UhpA-UhpB, VicK-VicR*) that facilitate rapid adaptation to environmental changes in response to external stimuli, (ii) quorum sensing (e.g., *luxS*) that mediates intercellular communication crucial for community coordination, (iii) biofilm formation (e.g., *bcsA*, *adrA, pemA, pelD-pelG*) that enhances surface adhesion and stability in environments, and (iv) flagellar assembly (e.g., *fliA-fliS, flhA, flhB, flhF, flgA-flgT*) that promoting motility and colonization capabilities (S1–S2 Tables). These regulatory pathways are hypothesized to function collaboratively with the biosurfactant synthesis mechanisms to maximize their functional output in response to environmental cues, thereby supporting processes like cell adhesion, motility, and community formation [65]. Specifically, the two-component system and quorum sensing provide mechanisms to coordinate actions for rapid responses to environmental cues, triggering bacterial motility and biofilm development [66–69]. Biofilm formation and flagellar assembly enhance the ability to colonize diverse ecological niches. Together, these findings support the potential for biosurfactant synthesis in strain 10A and, pending experimental validation, indicate that it may serve as a sustainable biosurfactant-producing system [65,70,71].

### Strain 10A is predicted to exhibit metabolic versatility

Pan-genomic analysis of available *Gallaecimonas* genomes revealed that *G. pentaromativorans* strain 10A and other members of the genus are predicted to share a number of core genes involved in pathways dealing with cellular and environmental information processing (Fig 3; S1–S2 Figs; S1–S2 Tables). These common cellular pathways are highly conserved in *Gammaproteobacteria*, encompassing critical functions such as cell growth and death, the citrate cycle, amino-acid metabolism, cell-wall metabolism, and the synthesis of peptidoglycan, a crucial component of bacterial cell walls [72,73]. Moreover, the conserved pathways for environmental information processing cover a wide array of functions vital for bacterial physiology and ecological interaction. For example, the osmotic stress response pathway, mediated by *MtrB-MtrA*, enables bacteria to regulate their internal osmolarity in response to fluctuating environmental conditions, which is particularly valuable given the presence of the genus in estuaries and intertidal environments [74]. Similarly, the regulation of membrane lipid fluidity by *DesK-DesR* is critical for preserving membrane integrity and function, especially in response to temperature fluctuations [75]. These capacities allow strain 10A and other *Gallaecimonas* spp. to adapt to varying environmental conditions, such as salinity and temperature, and maintain cellular function, likely contributing to the widespread presence of members of this genus across diverse environments.

Also conserved within the genus are genes that are predicted to be involved in capsule-synthesis pathways, including *ResC-ResD-ResB*, which play a vital role in providing protection against both host immune responses and environmental stresses [76]. However, capsules have not been reported in *Gallaecimonas*, suggesting that this pathway might have a different role or that the conditions needed for capsule formation have not yet been established. Interestingly, a conserved pathogenic cycle (map05111) found in both *Vibrio cholerae* and *Gallaecimonas* spp., suggests that both may be able to induce disease [77]. Ultimately, these conserved pathways highlight the sophisticated mechanisms that strain 10A and other isolates of *Gallaecimonas* spp. may employ for adaptation and proliferation in diverse environments.

Like other members in the genus *Gallaecimonas*, strain 10A is predicted to encode a diverse array of proteins involved in carbon, nitrogen, and sulfur cycling (Fig 3; S1–S2 Tables; S1–S3 Figs). Notably, the predicted capacity for methanol oxidation, likely for the acquisition of carbon and energy, suggests involvement in carbon cycling within marine ecosystems [78]. Moreover, genomic analysis revealed a complete nitrate-assimilation pathway and nitrate-assimilating genes (NAS), classifying strain 10A as a nitrate-assimilating bacterium (NAB) [78,79]. Additionally, the genomic makeup of strain 10A showcases both complete assimilatory and dissimilatory sulfate-reduction pathways, indicating chemosynthetic capabilities [80]. Sulfate-reducing bacteria (SRB) are important in sustaining the diversity and stability of marine bacterial communities [81]. Thus, strain 10A is predicted to have the capacity to play a key role in nitrogen, carbon, and sulfur cycling across a diverse range of environments.

### A predicted *higBA* stress-response toxin/anti-toxin system

Strain 10A is predicted to encode a *higBA* type II toxin-antitoxin (TA) system that is not found in other isolates of *Gallaecimonas* (Fig 3C; S1–S2 Tables). The system, consisting of *HigB-1* and *HigA-1*, responds to stress conditions, serves as a potential defense mechanism against stressors (e.g., cleaving foreign mRNA and encoding bacteriostatic toxins) [82]. Additionally, the TA system helps with adaptation to environmental challenges. For instance, in other gram-negative bacteria, such as *Pseudomonas aeruginosa,* the system regulates swarming and biofilm formation [82,83], and has been linked to survival strategies in fluctuating environmental conditions [82].

### The CRISPR-Cas system in members of the genus *Gallaecimonas*

The CRISPR-Cas system, an adaptive prokaryotic defense mechanism against foreign plasmids and viruses, was detected in *G. pentaromativorans* strain 10A [43] (Fig 4; S8 Fig; S4 Table). This discovery is notable, as CRISPR-Cas systems had not been reported for bacteria in the genus, *Gallaecimonas*. Genomic data from all five *Gallaecimonas* isolates, including strain 10A, revealed class 1 Type IE and IF CRISPR arrays and Cas clusters, common in gram-negative bacteria [84], in four out of the five genomes from *G. pentaromativorans*, *G. mangrovi*, and *G. xiamenensis* (Fig 4; S5–S8 Figs); *G. kandeliae* did not exhibit a full CRISPR-Cas system but CRISPR arrays were detected (S4 Fig). The Type IE systems in *G. pentaromativorans* strain 10A and *G. xiamenensis* strain 3-C-1 have a cascade, a multiprotein surveillance complex [84]. The Type IF system seen in *G. mangrovi* strain HK-28 and *G. pentaromativorans* strain CEE_131 (DSM 21945) have a *Csy* complex with *cas2* and *cas3* genes fused into a single open reading frame [84]. Notably, strain 10A possesses a complete Type IE CRISPR-Cas cassette with 95 spacers (Fig. 4; S8 Fig; S4 Table), suggesting a history of frequent encounters with foreign genetic elements, such as viruses.

### Strain 10A is morphologically similar to other isolates of *Gallaecimonas* spp

Similar to other isolates of *Gallaecimonas* spp., *G. pentaromativorans* strain 10A is rod-shaped, with cells about 0.5-μm wide and 2-μm long, although other members of the genus have been reported to range from 0.3- to 0.9-μm in width and 1- to 3-μm in length [3–5]. Based on the soft agar stab test, strain 10A appears to be motile, while preliminary TEM observations hint at the presence of a polar flagellum akin to that reported for *G. pentaromativorans* strain CEE_131 [1]; whereas, *G. xiamenensis* has an amphitrichous arrangement [3], and no flagella have been reported for *G. mangrove* [4] or *G. kandeliae* [5]. Furthermore, we identified the presence of putative genes (e.g., *PilB-PilV*) involved in type IV fimbriae (also known as type IV pili) synthesis in *G. pentaromativorans* strain 10A (S1 Table). This finding suggests that strain 10A may exhibit ‘twitching motility’, a process facilitated by the extension of long, thin fimbriae from the cell wall, which are involved in surface adherence and movement [85–87]. Type IV fimbriae are multifunctional structures and may play a variety of roles, including host attachment, biofilm formation, and/or potentially pathogenicity [87–91]. Interestingly, some of these type-IV-fimbriae-associated genes (e.g., *PilS-PilR*) were also identified as core genes shared among all four *Gallaecimonas* species described to date, highlighting their potentially important role within the genus.

*Gallaecimonas* colonies, including strain 10A, are consistently characterized as being smooth, circular, convex, colorless to gray colored, and 0.1- to 3-mm in diameter [1,3–5].

### Members of the genus *Gallaecimonas* are widespread across environments

Bacteria in the genus *Gallaecimona*s demonstrate remarkable ecological versatility, as evidenced by their widespread presence across different environments at all latitudes (Fig 5; S5 Table). Members of the genus have primarily been reported from marine environments, aligning with isolations from a crude oil-degrading consortium in seawater [3], mangrove sediments [4,5], and intertidal sediments [1]. However, environmental sequencing data indicate that their range also extends to soils, estuaries, rivers, and the cryosphere (Fig 5; S5 Table). Thus, bacteria in the genus exist across a wide range of salinities, temperatures, and environmental conditions. In fact, members of the genus have been grown at 10–45 °C, pH 5–10, and NaCl concentrations from 0 to12% [1,3–5].

The species-specific biogeography of *Gallaecimonas* is limited in the current investigation because the GBIF 16S rRNA gene database only includes samples from 2003 to 2019, which does not reflect recent taxonomic updates and overlooks species such as *G. mangrovi* and *G. kandeliae*. Similarly, *G. pentaromativorans*, including strain 10A, was only detected on two occasions in marine sediment and seawater. Such a low occurrence of *G. pentaromativorans* in natural environments might reflect its host-associated nature (e.g., strain 10A), suggesting that we need to search for them in animal microbiomes (e.g., oysters). Moreover, the distribution of *G. pentaromativorans* strain 10A in global ecosystems also requires more sampling effort and accurate taxonomic assignment using the most current 16S rRNA gene database (e.g., the future database with strain 10A included).

## Conclusions

Here, we present the first complete genome published for the species, *Gallaecimonas pentaromativorans*. The isolation and genomic characterization of *G. pentaromativorans* strain 10A from Pacific oyster, alongside comparative genomics with other isolates, demonstrates the remarkable genetic potential of strain 10A, and of members of the genus, more broadly. As well, by mining environmental data, we demonstrate the widespread distribution of the genus globally and across environments (e.g., seawater, sediment, and oysters). Functionally, genome annotations suggest that strain 10A may contribute to environmentally relevant processes such as carbon cycling and nitrogen and sulfur metabolism, with potential for nitrate and sulfate reduction. Genes associated with biosurfactant synthesis and the degradation of xenobiotics, including PAHs, also point to a possible role in bioremediation and biosurfactant production. However, these functional predictions remain hypothetical and require experimental validation. Future work is essential to confirm these capabilities and clarify the ecological roles and biotechnological relevance of strain 10A. Moreover, strain 10A was isolated from dead or dying Pacific oysters during a mass mortality event on a commercial farm. Whether strain 10A is an intrinsic member of the oyster microbiome or a secondary colonizer that proliferates on oyster carcasses remains unclear and warrants further investigation. Notably, there is no evidence linking strain 10A to the oyster die-off in BC, which has instead been associated with an RNA virus [92].

## Supporting information

S1 Fig Functional maps of *Gallaecimonas.* Functional genomic maps for the five isolates of *Gallaecimonas* spp. for which full genome sequences are available (A) *G. pentaromativorans* strain CEE_131 (Leibniz Institute DSMZ culture collection ID: DSM 21945); (B) *G. xiamenensis* strain 3-C-1; (C) *G. pentaromativorans* strain 10A; (D) *G. kandeliae* strain Q10; (E) *G. mangrovi* strain HK-28) made with FuncTree v.0.8.4. Node color is depicted as follows from the outermost to innermost rings of the map: yellow (only for strain 10A) denotes KEGG Orthology (ko); red signifies KEGG Module; green indicates KEGG Pathways; light blue represents biological processes; dark blue represents biological categories. The position on the circle represents category: I. Human Diseases, II. Metabolism, III. Genetic Information Processing, IV. Environmental Information Processing, V. Cellular Processes, VI. Organismal Systems. The node size corresponds to the value of the standard deviation of the ko’s relative abundance assigned to that function.
(TIF)

S2 Fig Comparative genomic features of *Gallaecimonas* spp. Venn diagrams illustrating the distribution of (A) CAZy, (B) COG profiles, (C) EC ids, (D) eggNOG Ogs, (E) GO terms, (F) PFAMs (i.e., protein family), (G) KEGG ko profiles, (H) KEGG Module profiles, (I) KEGG Pathway profiles among genomes of *Gallaecimonas* spp. (I. *G. pentaromativorans* strain 10A; II. *G. pentaromativorans* strain CEE_131 (Leibniz Institute DSMZ culture collection ID: DSM 21945); III. *G. xiamenensis* strain 3-C-1; IV. *G. kandeliae* strain Q10; V. *G. mangrovi* strain HK-28) according to eggNOG-mapper.
(TIF)

S3 Fig PAHs and xenobiotics degradation potential of *G. pentaromativorans* strain 10A. Functional map of the polycyclic aromatic hydrocarbons (PAHs) and xenobiotics degradation KEGG ko profiles with over 50% coverage for module and pathway made with FuncTree v.0.8.4. The outermost to innermost rings are as follows: yellow represents KEGG Orthology (ko), and green represents KEGG Pathways. Node size corresponds to the value of the standard deviation of the KEGG profile’s relative abundance assigned to that function.
(TIF)

S4 Fig CRISPR-Cas analysis of *G. kandeliae* strain Q10. Genomic map of *G. kandeliae* strain Q10, annotated with the clustered regularly interspaced short palindromic repeats and spacers (CRISPR arrays), CRISPR-associated (Cas) proteins and clusters, and putative coding sequences (CDSs). Annotations are based on sequence similarities to known Cas proteins using HMM protein profiles and identified using CRISPRCasFinder.
(TIF)

S5 Fig CRISPR-Cas analysis of *G. mangrovi* strain HK-28. See legend of S4 Fig for details.
(TIF)

S6 Fig CRISPR-Cas analysis of *G. pentaromativorans* strain CEE_131. See legend of S4 Fig for details.
(TIF)

S7 Fig CRISPR-Cas analysis of *G. xiamenensis* strain 3-C-1. See legend of S4 Fig for details.
(TIF)

S8 Fig CRISPR-Cas analysis of *G. pentaromativorans* strain 10A. See legend of S4 Fig for details.
(TIF)

S1 Table Functional annotation of genes in *Gallaecimonas pentaromativorans* strain 10A. Data consists of predicted seed ortholog (i.e., gene family), e-value, score, eggNOG OGs, max annotation level, COG category, description, preferred name, GOs, EC, KEGG profile data (ko, Pathway, Module, Reaction, rclass, TC), BRITE, CAZy, BiGG Reaction, and PFAMs (i.e., protein family) using eggNOG-mapper.
(XLSX)

S2 Table Metadata for comparative genomics. The eggNOG-mapper generated data of (i) common KEGG Ortholog (ko) profiles for *Gallaecimonas* spp., (ii) common ko profiles for *G. pentaromativorans*, and (iii-vii) unique ko profiles for each *Gallaecimonas* spp.
(XLSX)

S3 Table Hydrocarbon degradation–related genes identified in the genome of *G. pentaromativorans* strain 10A. (XLSX)

S4 Table CRISPR repeat and spacer sequences detected in the genome of *G. pentaromativorans* strain 10A. (XLSX)

S5 Table Metadata for analysis of the distribution of *Gallaecimonas* across geolocations and habitats. Data sourced from the GBIF Database and cleaned to include gbifID, occurrenceID, materialSampleID, eventID, sampleSizeValue, sampleSizeUnit, continent, waterBody, genus, specificEpithet, depth, ScientificName, and environment biome, feature and material.
(XLSX)
