# Supplementary figures and images for: The genome of Gallaecimonas pentaromativorans strain 10A, isolated from a Pacific oyster, sheds light on an environmentally widespread genus with remarkable metabolic potential
Source: PLoS One. 2025 Oct 21;20(10):e0334406. doi: 10.1371/journal.pone.0334406 (PMC12539734; doi:10.1371/journal.pone.0334406)

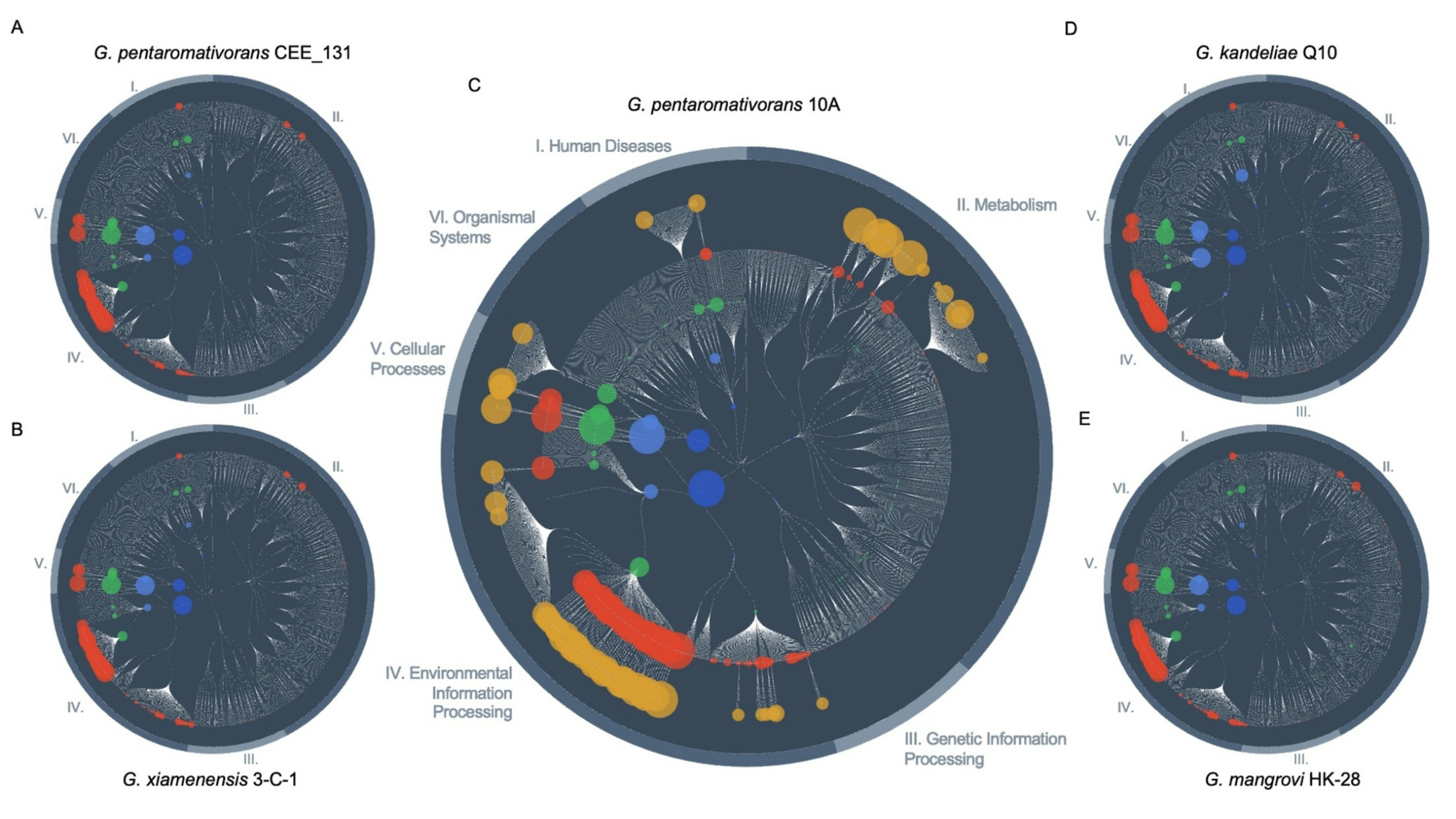

Supplement: S1 Fig — Functional genomic maps for the five isolates of Gallaecimonas spp. for which full genome sequences are available (A) G. pentaromativorans strain CEE_131 (Leibniz Institute DSMZ culture collection ID: DSM 21945); (B) G. xiamenensis strain 3-C-1; (C) G. pentaromativorans strain 10A; (D) G. kandeliae strain Q10; (E) G. mangrovi strain HK-28) made with FuncTree v.0.8.4. Node color is depicted as follows from the outermost to innermost rings of the map: yellow (only for strain 10A) denotes KEGG Orthology (ko); red signifies KEGG Module; green indicates KEGG Pathways; light blue represents biological processes; dark blue represents biological categories. The position on the circle represents category: I. Human Diseases, II. Metabolism, III. Genetic Information Processing, IV. Environmental Information Processing, V. Cellular Processes, VI. Organismal Systems. The node size corresponds to the value of the standard deviation of the ko’s relative abundance assigned to that function. (TIF) [file pone.0334406.s001.tif]

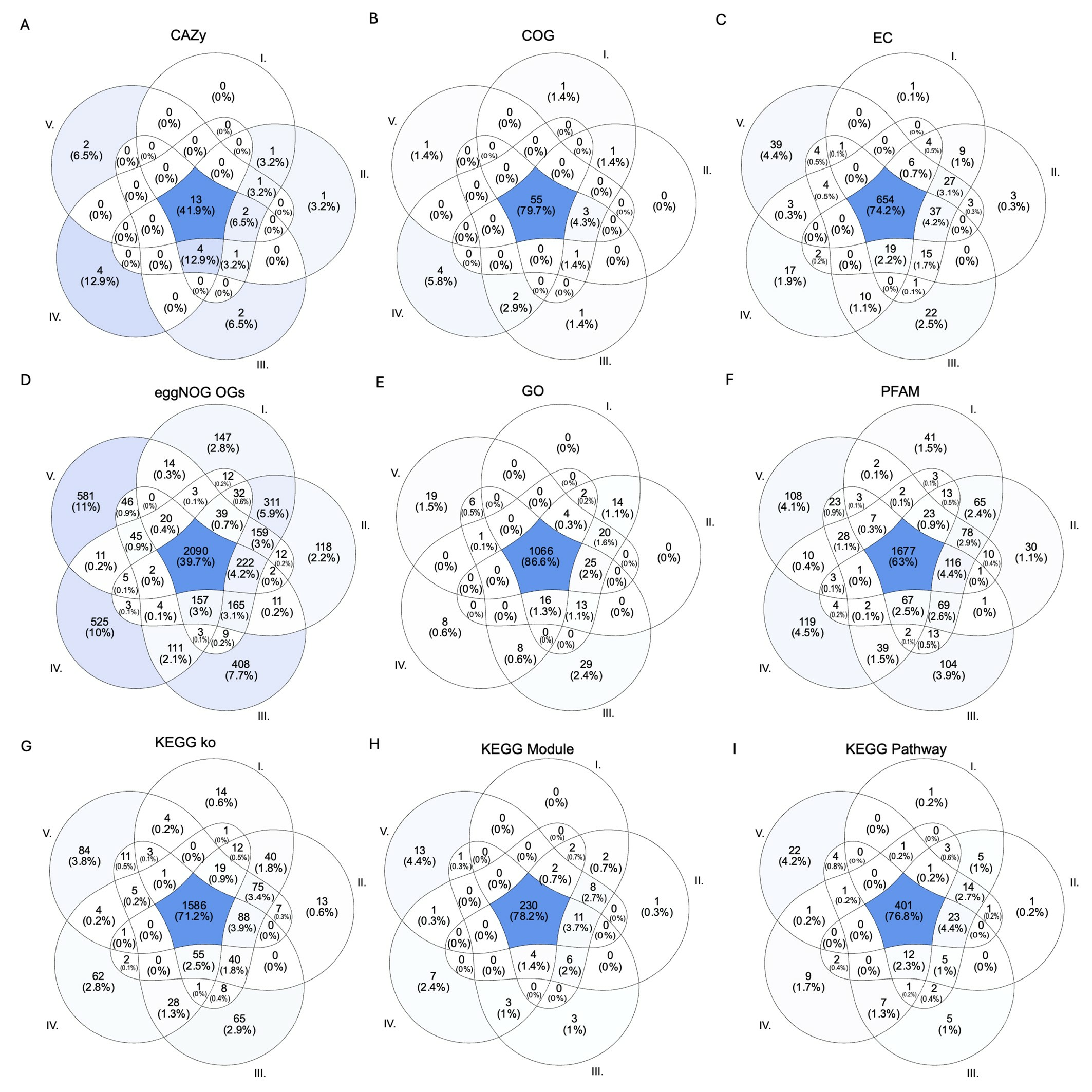

Supplement: S2 Fig — Venn diagrams illustrating the distribution of (A) CAZy, (B) COG profiles, (C) EC ids, (D) eggNOG Ogs, (E) GO terms, (F) PFAMs (i.e., protein family), (G) KEGG ko profiles, (H) KEGG Module profiles, (I) KEGG Pathway profiles among genomes of Gallaecimonas spp. (I. G. pentaromativorans strain 10A; II. G. pentaromativorans strain CEE_131 (Leibniz Institute DSMZ culture collection ID: DSM 21945); III. G. xiamenensis strain 3-C-1; IV. G. kandeliae strain Q10; V. G. mangrovi strain HK-28) according to eggNOG-mapper. (TIF) [file pone.0334406.s002.tif]

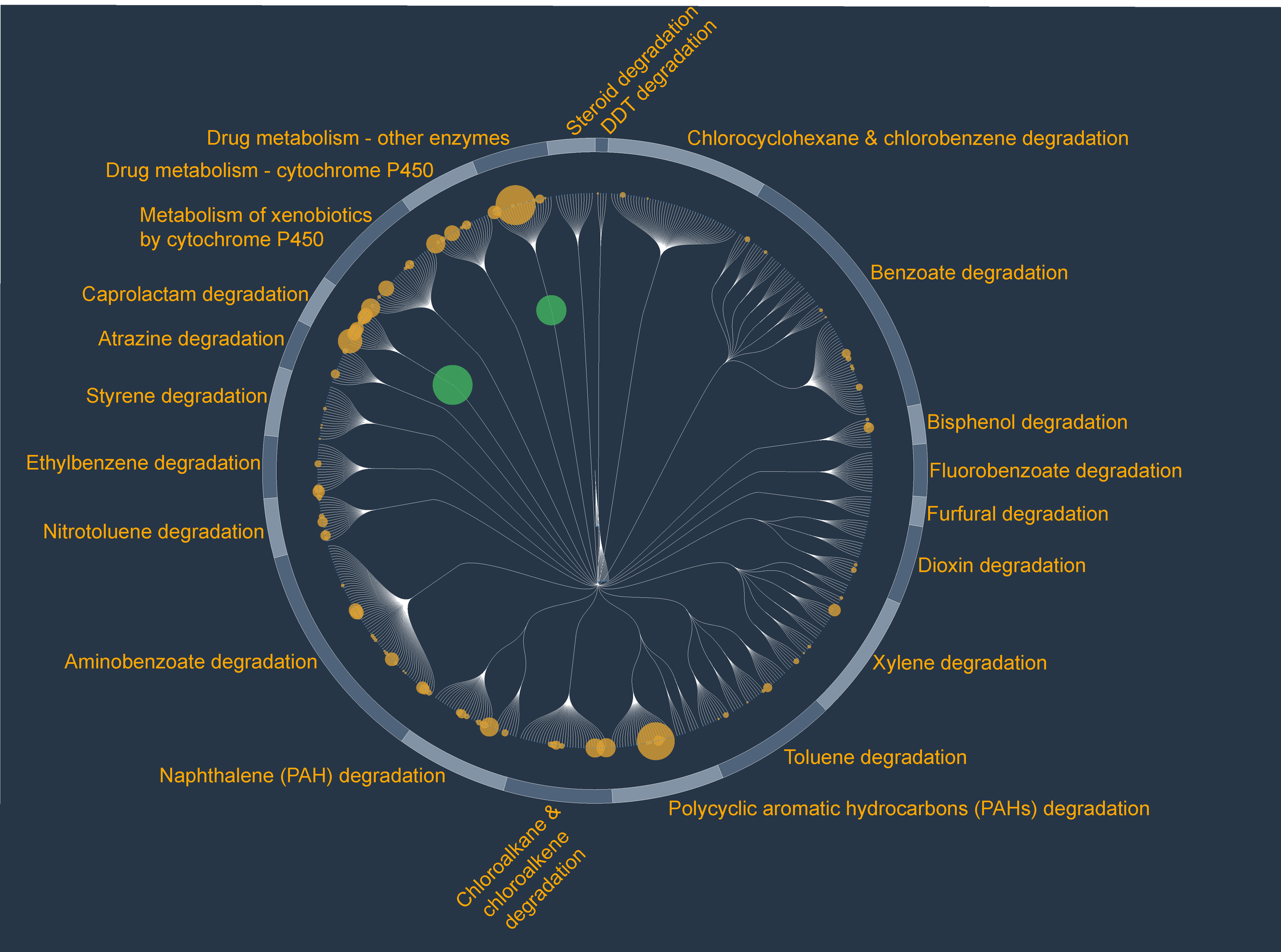

Supplement: S3 Fig — Functional map of the polycyclic aromatic hydrocarbons (PAHs) and xenobiotics degradation KEGG ko profiles with over 50% coverage for module and pathway made with FuncTree v.0.8.4. The outermost to innermost rings are as follows: yellow represents KEGG Orthology (ko), and green represents KEGG Pathways. Node size corresponds to the value of the standard deviation of the KEGG profile’s relative abundance assigned to that function. (TIF) [file pone.0334406.s003.tif]

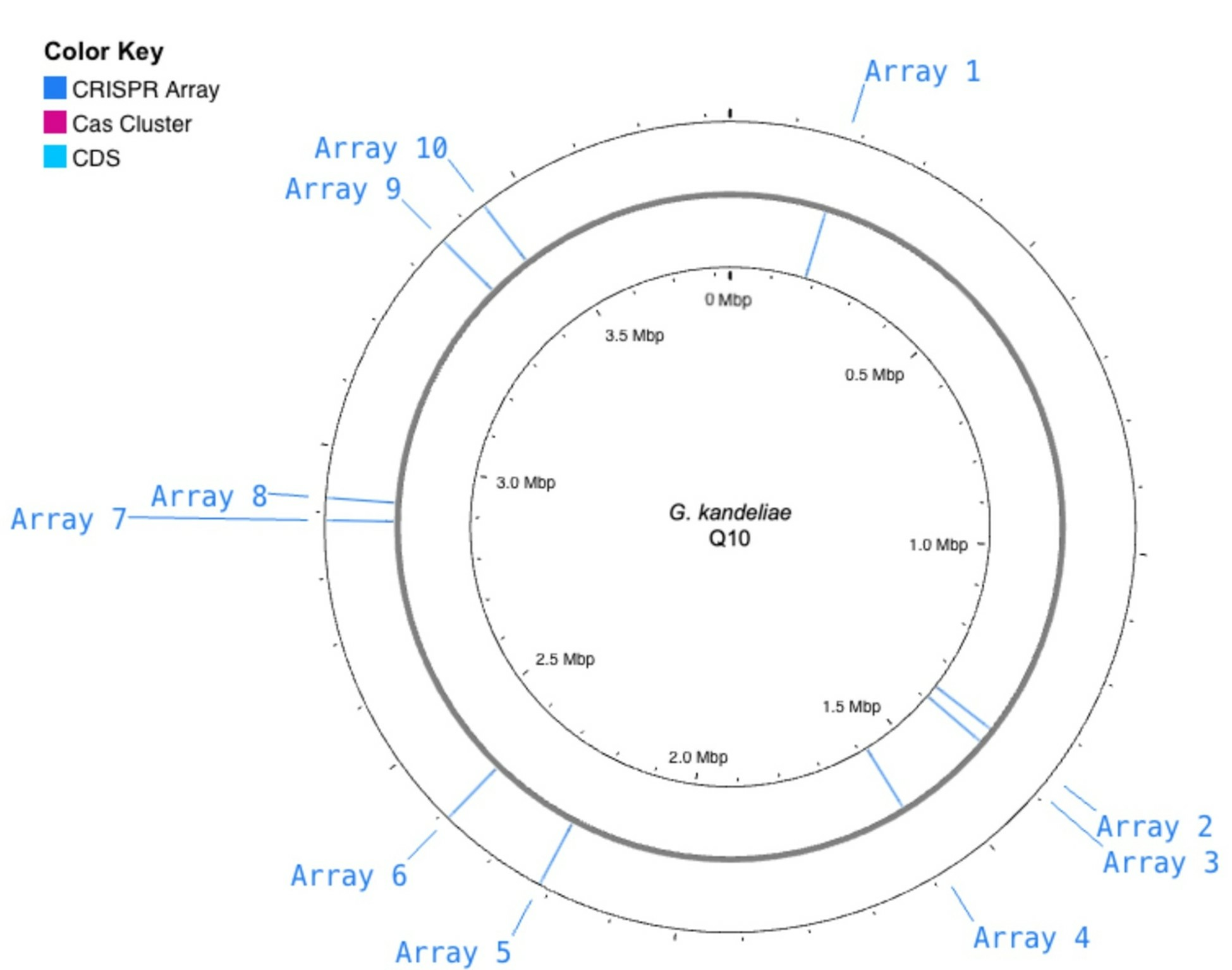

Supplement: S4 Fig — Genomic map of G. kandeliae strain Q10, annotated with the clustered regularly interspaced short palindromic repeats and spacers (CRISPR arrays), CRISPR-associated (Cas) proteins and clusters, and putative coding sequences (CDSs). Annotations are based on sequence similarities to known Cas proteins using HMM protein profiles and identified using CRISPRCasFinder. (TIF) [file pone.0334406.s004.tif]

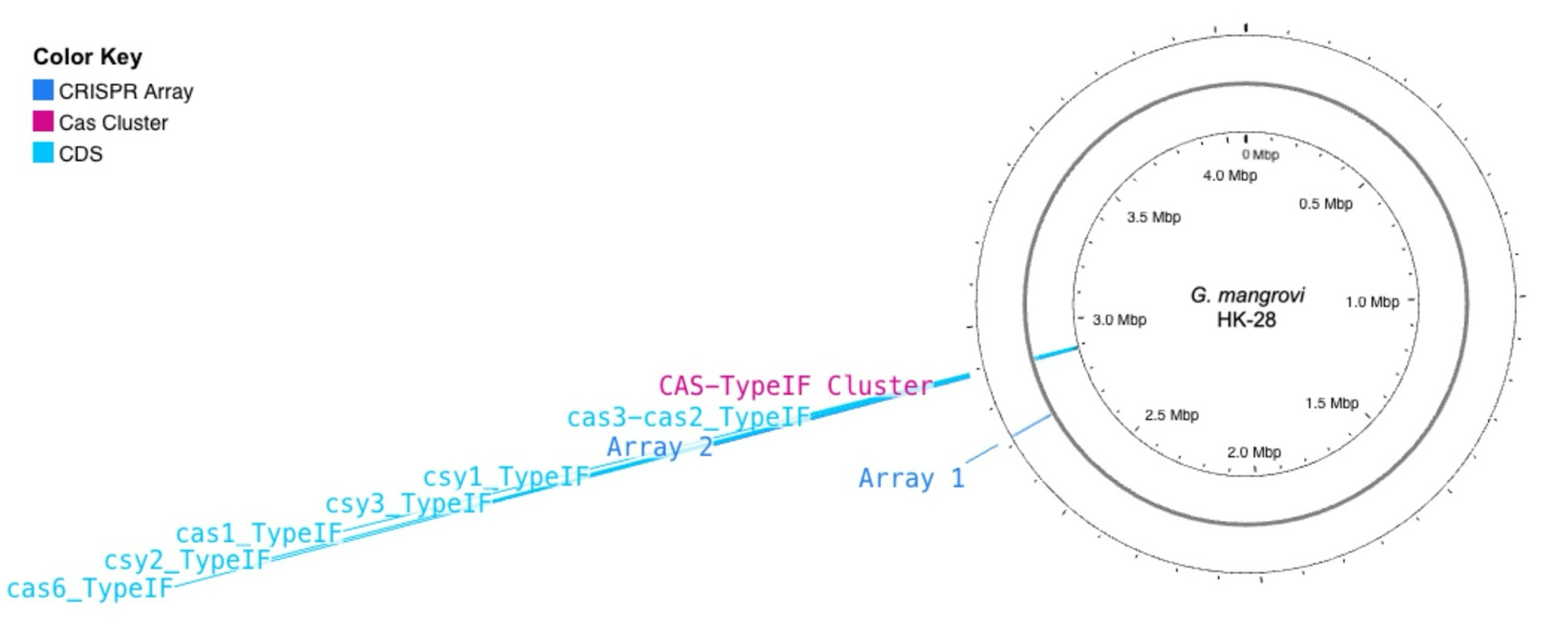

Supplement: S5 Fig — See legend of S4 Fig for details. (TIF) [file pone.0334406.s005.tif]

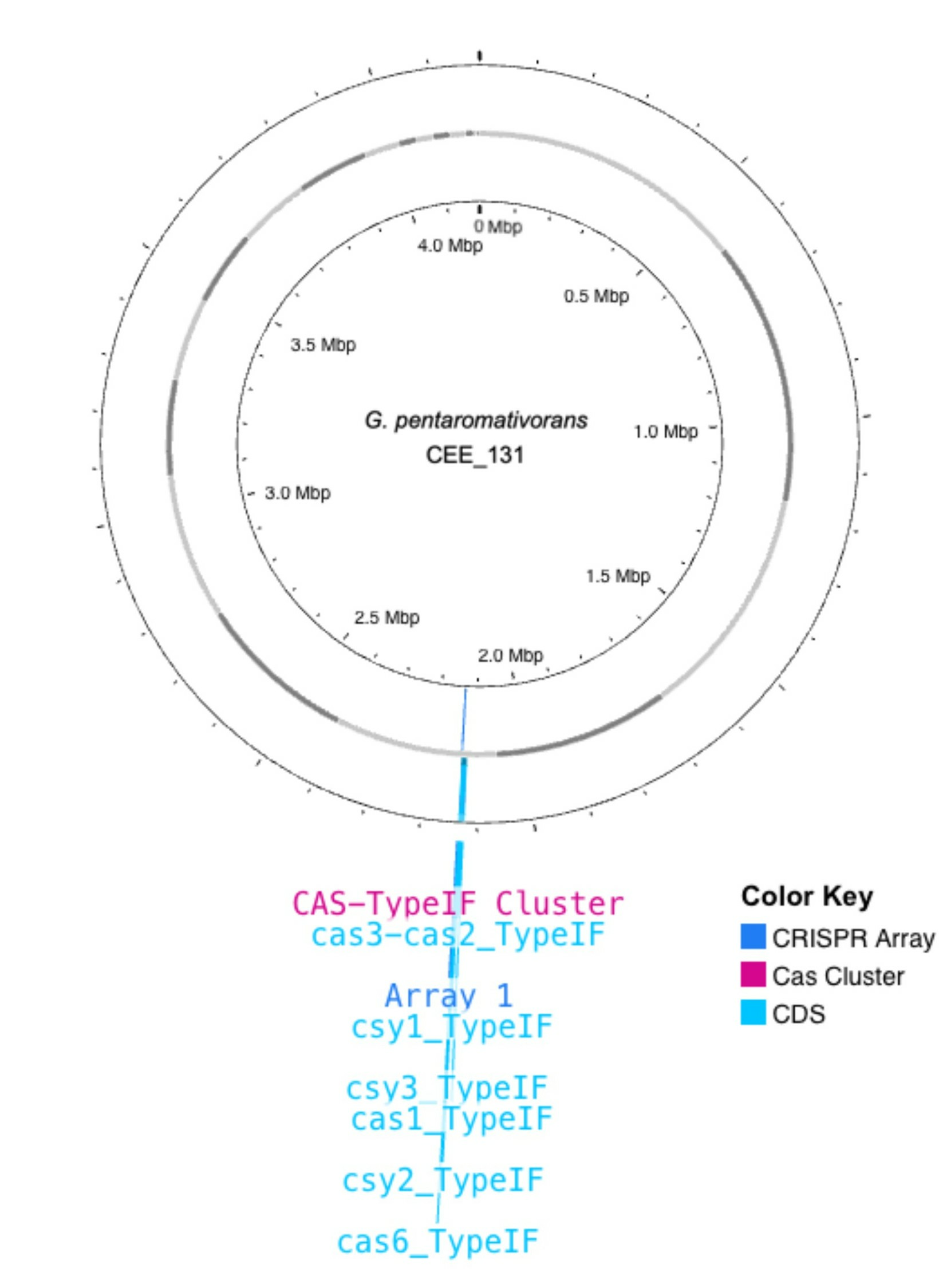

Supplement: S6 Fig — See legend of S4 Fig for details. (TIF) [file pone.0334406.s006.tif]

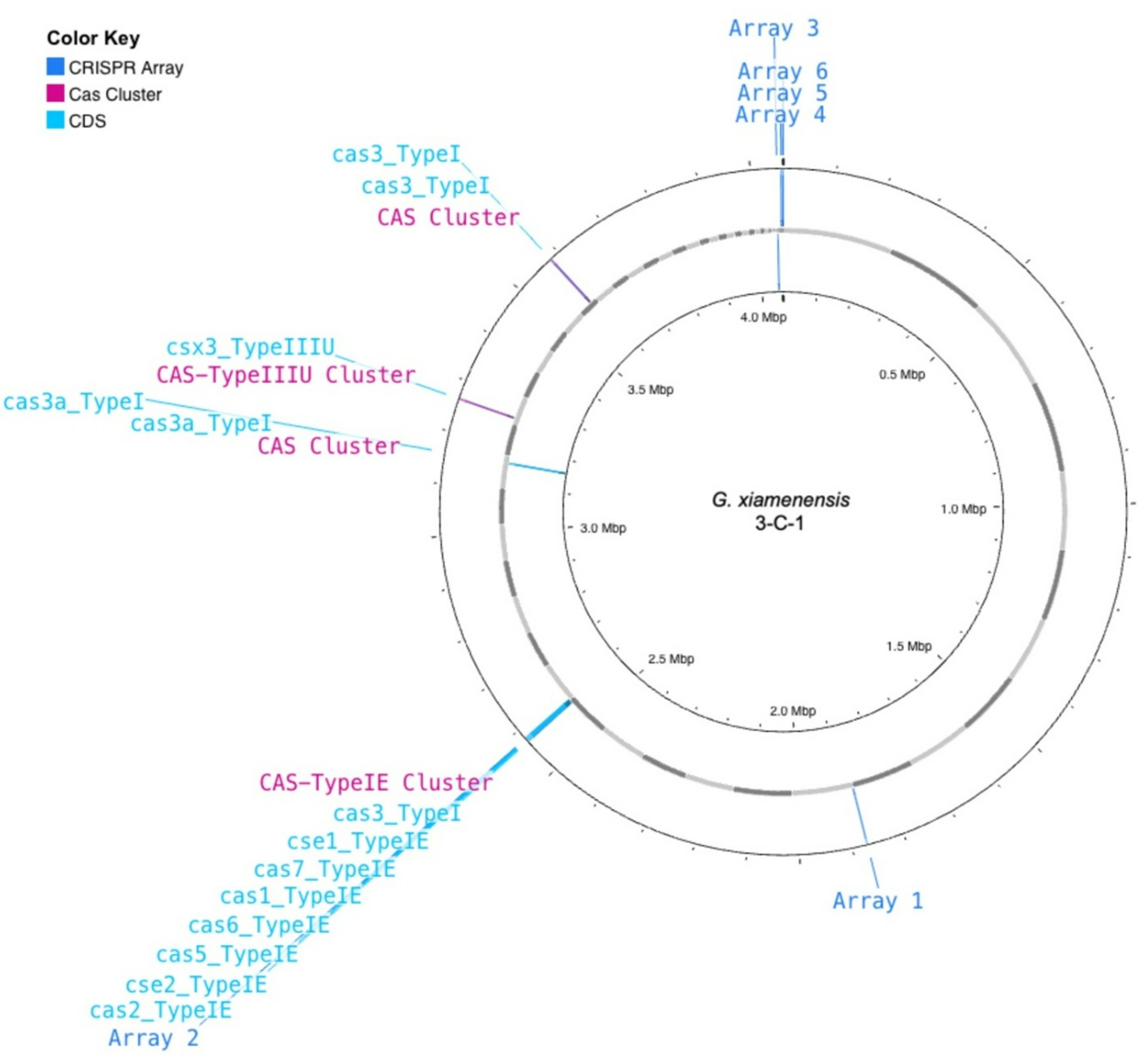

Supplement: S7 Fig — See legend of S4 Fig for details. (TIF) [file pone.0334406.s007.tif]

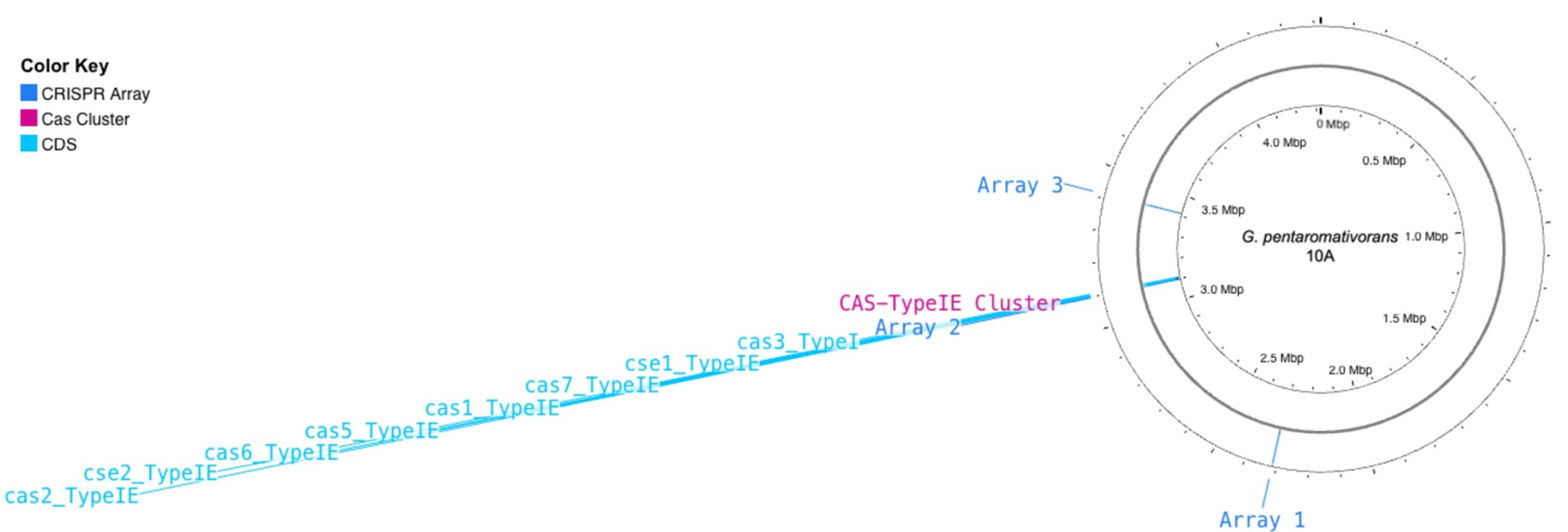

Supplement: S8 Fig — See legend of S4 Fig for details. (TIF) [file pone.0334406.s008.tif]
